# Evaluating the molecular function of zebrafish Anoctamin 1a

**DOI:** 10.17912/micropub.biology.001706

**Published:** 2025-07-30

**Authors:** Amanda Dimatteo, Nicole Stango, Adam Rich, Tara Sweet

**Affiliations:** 1 Biology, SUNY Geneseo, Geneseo, New York, United States; 2 Biology, SUNY Brockport, Brockport, New York, United States; 3 Biology-Psychology, SUNY Geneseo, Geneseo, New York, United States

## Abstract

Calcium-activated chloride channels (CaCCs) regulate key physiological processes like epithelial secretion, sensory transduction, gastrointestinal pacemaking, and muscle contraction. Zebrafish ( *Danio rerio* ) are genetically similar to humans, easily manipulated, and are a valuable model organism. We cloned zebrafish Anoctamin 1, a putative CaCC gene, expressed it in mammalian cells, and confirmed its function via halide flux assays. The channel conducted anions and responded to calcium, verifying its role as a CaCC. These findings confirm the functionality of zebrafish CaCCs and lay the groundwork to use the organism to model human physiology and disease.

**Figure 1. Heterologous Ano1 expression in CHO-K1 cells:**
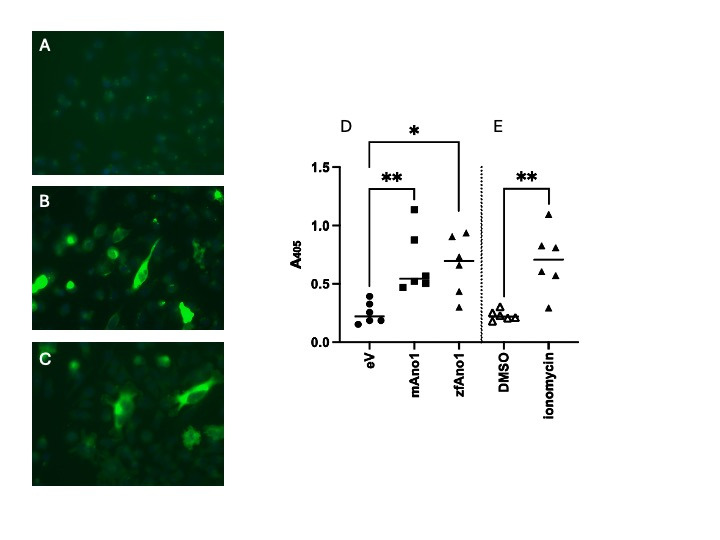
Left: Transfection of plasmids containing (A) vector, (B) mouse Ano1, or (C) zebrafish Ano1. Images were merged and taken at 40X with Ano1 immunoreactivity under the green filter and DAPI staining under the blue filter. Right: (D) Absorbance at 405 nm following a modified Sandell-Kolthoff reaction. Lysates from CHO-K1 cells transfected with empty vector (mean: 0.250, STD: 0.094, n = 6), mouse Ano1 (mean: 0.679, STD: 0.286, n = 6), or zebrafish Ano1 (mean: 0.662, STD: 0.234, n = 6) with the efflux assay run in the presence of 100 µM ionomycin. A Kruskal Wallis test revealed a statistically significant difference in absorbance between groups (H(3) = 10.15, p = 0.002). Dunn’s Post test for multiple comparisons showed significant differences in absorbance between eV and mouse Ano1 (p = 0.01), and between eV and zebrafish Ano1 (p = 0.01). (E) Absorbance at 405 nm following a modified Sandell-Kolthoff reaction. Lysates from CHO-K1 cells transfected with zebrafish Ano1 with the efflux assay run in the presence of 100 µM of ionomycin, or vehicle. An independent T-test showed a significant difference in scores between the two groups, t(10) = 4.16, p = .001. Ionomycin (mean: 0.701, 95% C.I. =0.414 - 0.988, n = 6) had a higher OD405 as compared to the vehicle (mean: 0.230, 95% C.I. = 0.185 - 0.275, n = 6), indicating a calcium dependence of the flux.

## Description

Anoctamins (TMEM16 proteins) are a diverse family of calcium-activated transmembrane proteins broadly expressed in various tissues. Some members of this family act as ion channels that conduct ions in response to elevated intracellular calcium levels, while others serve as phospholipid scramblases that redistribute lipids across the membrane bilayer. These proteins regulate processes such as membrane potential, cellular excitability, and phospholipid distribution. Specific members like ANO2 (TMEM16B), expressed in photoreceptors and olfactory neurons, are crucial for sensory transduction (Dauner et al., 2013; Stephan et al., 2009). ANO6 (TMEM16F) functions as a scramblase in blood cells and is essential for proper blood coagulation (H. Yang et al., 2012).

Anoctamin 1 (ANO1, also called TMEM16A or DOG1), the founding member of this family, functions as a calcium-activated anion channel that conducts chloride ions across membranes (Caputo et al., 2008; Schroeder et al., 2008; Y. D. Yang et al., 2008). ANO1 is expressed in diverse cell types including epithelial cells, interstitial cells of Cajal (ICCs), smooth muscle, skeletal muscle, and sensory neurons (Dayal et al., 2019; Griffin et al., 1982; Hwang et al., 2009). In epithelial tissues, ANO1 regulates chloride secretion vital for fluid and electrolyte balance in organs such as the lungs, intestines, and salivary glands (Centeio et al., 2021; Ousingsawat et al., 2024; Romanenko et al., 2010). In ICCs, ANO1 contributes to rhythmic electrical oscillations that drive gastrointestinal motility (Hwang et al., 2009). In smooth and skeletal muscle, it influences contractility and membrane excitability, impacting vascular tone and muscle function (Dayal et al., 2019; Heinze et al., 2014). ANO1 also participates in sensory transduction related to pain and itch perception (Huang et al., 2013; Kim et al., 2024). Dysregulation or mutation of ANO1 has been associated with conditions such as gastrointestinal dysmotility, vascular disorders, and various cancers, underlining its importance in health and disease (Al Sharie et al., 2023; Li et al., 2022; Pinard et al., 2023).

The zebrafish (Danio rerio) is a valuable vertebrate model for studying human physiology and disease due to its genetic similarity to humans—approximately 70% of human genes have zebrafish orthologs. Zebrafish embryos develop externally, are transparent during early stages, and are amenable to genetic manipulation techniques like CRISPR/Cas9, facilitating the creation of disease models. Their rapid development, high reproductive rate, and suitability for high-throughput screening make them ideal for exploring disease mechanisms and therapeutic interventions across a range of conditions, including cancer, cardiovascular, neurodegenerative, and congenital diseases.

To investigate zebrafish Ano1 function and its relevance to vertebrate physiology, we cloned the putative *ano1* gene from zebrafish larvae, expressed it in a mammalian cell system, and assessed its functional properties.

*Cloning and Sequence Analysis of Zebrafish ano1*

The zebrafish *ano1a* gene was cloned into the pcDNA3.1+ mammalian expression vector and sequenced via Sanger sequencing. Multiple overlapping reads confirmed high similarity to the predicted Danio rerio anoctamin 1 X2 isoform on chromosome 7 (GenBank XM_068222213.1). Sequence comparison revealed 13 differences: 11 silent nucleotide changes and two non-silent substitutions at positions 94 and 222. These resulted in amino acid changes: glutamate replacing aspartate at position 14 and glycine replacing glutamate at position 57.

Protein sequence analysis showed high conservation with human and mouse ANO1 orthologs, especially within the transmembrane regions TM3 to TM10. The critical sites for calcium binding were conserved, indicating potential functional similarity (Le et al., 2021). However, the zebrafish isoform lacked exons previously referred to as b, c, and d which alter the channel’s calcium sensitivity, voltage dependence and gating kinetics (Ferrera et al., 2009; Strege et al., 2017; Xiao et al., 2011).

*Expression of Zebrafish Ano1 in Mammalian Cells*

CHO-K1 cells were transfected with the cloned zebrafish Ano1a construct, mouse Ano1, or empty vector to assess heterologous protein expression. Immunostaining using anti-human ANO1 antibodies revealed plasma membrane and cytoplasmic localization of zebrafish Ano1, comparable to mouse Ano1 expression patterns. Figure 1, panel A shows no signal was detected in empty vector controls, confirming specificity and successful expression of zebrafish Ano1a.

*Functional Characterization Using Iodine Flux Assay*

To determine whether zebrafish Ano1a functions as a CaCC similar to its mammalian counterparts (Caputo et al., 2008; Maduke et al., 2000; Y. D. Yang et al., 2008), a colorimetric iodine flux assay was employed (Tang & Wildey, 2004). In this assay, iodine efflux catalyzes the reduction of cerium (Ce), from a yellow Ce4+ to colorless Ce3+, allowing quantification via absorbance. Transiently transfected cells loaded with iodine were treated with calcium ionophore ionomycin to activate Ano1 channels, followed by cell lysis and analysis (Liu & Hermann, 1978).

CHO-K1 cells expressing zebrafish or mouse Ano1 showed significantly higher absorbance compared to empty vector controls, indicating successful iodine efflux and functional anion channel activity. This efflux was calcium-dependent, as cells treated with ionomycin exhibited greater absorbance than vehicle-treated cells. These findings confirm that zebrafish Ano1a functions as a calcium-activated halide channel.

The observed 2- to 3-fold increase in absorbance following ionomycin treatment is consistent with activation of a calcium-dependent anion channel, although it may different in magnitude than changes previously reported for GABA _A_ receptors by Tang and Wildey (2004). This difference likely reflects distinct channel gating mechanisms: GABA _A_ is a ligand-gated chloride channel directly activated by GABA, whereas Ano1 is a CaCC that responds to elevated intracellular calcium, which ionomycin induces both directly and indirectly through calcium-induced calcium release. Additionally, greater variability was observed across biological replicates in this study. While the use of transiently transfected CHO-K1 cells may contribute to this variation, other factors such as initial cell density, growth or death rates, calcium handling, expression levels, or cell detachment (potentially due to solution exchange) are also likely contributors. Ongoing work is focused on identifying and minimizing these sources of variability to improve assay consistency and reliability.

This study demonstrates that zebrafish Ano1a is a functional CaCC, capable of conducting anions in a calcium-dependent manner, consistent with its role in other vertebrates such as Xenopus, mouse, and human (Caputo et al., 2008; Schroeder et al., 2008; Y. D. Yang et al., 2008). These results provide foundational evidence for the physiological relevance of zebrafish Ano1a and suggest potential roles in processes like osmoregulation, sensory perception, and gastrointestinal function.

For example, zebrafish and other teleost fish must adapt to varying salinity levels during freshwater and seawater transitions (Evans et al., 2005). In euryhaline mummichogs, ano1 expression in gill epithelium increases with salinity exposure, supporting its role in chloride ion transport essential for osmoregulation (Breves et al., 2024). Given the similarity between mummichog and zebrafish Ano1, a similar mechanism may occur in zebrafish.

CaCCs are also important in sensory systems such as olfaction and taste (Dibattista et al., 2024). Zebrafish rely on their lateral line system—a mechanosensory organ detecting water movement and vibration—crucial for navigation, prey detection, and predator avoidance. Although Ano2 is known to be expressed in neuromast hair cells, Ano1 may also contribute to CaCC activity in this sensory network, potentially influencing behavior and environmental response (Lunsford et al., 2023).

In the gastrointestinal system, ICCs generate slow-wave electrical rhythms coordinating gut motility (Huizinga et al., 2014; Sanders, 2019). In mammals, ANO1 is essential for this pacemaker activity, and mutations are linked to dysmotility disorders (Al Sharie et al., 2023). Zebrafish possess ICC-like cells expressing Ano1, suggesting a conserved role in regulating digestive tract function (Uyttebroek et al., 2013). Future research may reveal how Ano1 dysfunction impacts zebrafish gut motility, offering insights applicable to human gastrointestinal disorders.

Recent studies show that Ano1 influences skeletal muscle function in zebrafish by accelerating action potentials. This complements our findings and prior research showing that reducing Ano1 expression via siRNA diminishes CaCC currents in muscle cells (9). These data suggest Ano1 is critical for muscle excitability and contraction, consistent with its role in mammalian muscle physiology.

In summary, we cloned and expressed zebrafish Ano1a, confirming its function as a CaCC through iodine flux assays. Zebrafish Ano1 shares conserved structural and functional characteristics with mammalian counterparts, validating its use in modeling ANO1-associated physiological and pathological processes. This study lays the groundwork for leveraging zebrafish as a model system to explore Ano1's role in sensory biology, muscle function, osmoregulation, and gastrointestinal motility, with implications for understanding human disease mechanisms and potential treatments.

## Methods

Tissue was harvested from a pool of fifteen anesthetized unsexed 7 days post fertilization larvae generated from a multi-fish breeding of the ab/ab line housed in the lab of Dr. Rich. Larvae that did not show age-appropriate development were excluded. Previous experiments using this number of larvae produced sufficient RNA to amplify a segment of the ano1 by PCR. Wild-type zebrafish stocks, originally obtained from the Zebrafish International Resource Center, were housed and maintained in our facility at SUNY Brockport. All procedures were conducted following established protocols and in full compliance with the SUNY Brockport IACUC guidelines (Approval Number: Tissue Collection PROTO202400005) (Rich et al., 2007).

*Cloning of zebrafish ano1*

Tissue was homogenized in Qiazol lysis reagent (Qiagen, 79306). RNA was phase separated from the lysate by addition of chloroform-isoamyl alcohol (Sigma, C0549) and precipitated with isopropanol (Sigma, I9516). From the RNA, cDNA was prepared using a verso cDNA synthesis kit with oligo dT primers per manufacturer’s instructions (Thermo Scientific, AB-1453). Primers were designed based on the published zebrafish genomic sequence (GRCz11) to capture the isoforms overlapping coding regions and incorporate cloning restriction enzyme cut sites. The sequence of the forward primer was cgacacacaggtacCGCTGTACGGTCAACCTGG and the sequence of the reverse primer was cagacttctaGATTACGCTTGTGAACTGGGCCGCGAGCGG. cDNA was amplified with high- fidelity polymerase Pfusion Hotstart II (Thermo Scientific, F-549L), cleaned, digested and cloned into expression vectors pcDNA3.1+ (Invitrogen, V79020). The nucleotide sequence the insert was determined by Sanger Sequencing (Genewiz, South Plainfield, NJ), and the full-length sequence has been uploaded to Genbank (PQ62605).

*Sequence Analysis of ano1*

Using the clustal omega algorithm (available through https://www.ebi.ac.uk/jdispatcher/), the cloned zebrafish sequence was aligned with zebrafish predicted sequences (X1: XM_009303471.3, X2: XM_068222213.1, X3: XM_009303473.3, X4: XM_017356841.2). The zebrafish nucleotide sequence was translated to a protein sequence using Expasy (available at https://web.expasy.org/translate/) and aligned with the clustal omega algorithm to human Anoctamin 1a (NP_060513.5) and mouse Anoctamin 1 “a” also referred to as anoctamin-1 isoform 2 (NP_001229278.2). These isoforms were chosen as known sequences and for consistency with previously published literature (Le et al., 2021), but the authors note zebrafish ano1 shows stronger alignment to the hypothetical human isoform ANO1 X6 (XP_054224207.1) largely because there is more available sequence that aligns at the N-termini.

*Cell culture*

CHOK1 cells (ATCC CAT# CCL-61) were grown in F12-K medium (ATCC 30-2004) containing 10% FBS (Gibco, A52095) and 1X Penicillin-Streptomycin-Glutamine mixture (Gibco, 10378). They were cultured in a tissue culture incubator at 37 °C with 5% CO2. For imaging, 2 ml cells with a density of ~125,000/ml were dispensed into 12 well-containing D-lysine-coated (Gibco, A38904) glass coverslips. After overnight culture, cells were transfected using lipofectamine 2000 per manufacturer’s instructions (Invitrogen, 11668019). To evaluate expression of ano1, cells were transfected with an empty pcDNA3.1+ plasmid containing no insert, a pCMV6 plasmid containing mouse Ano1 (NM_178642, Cat # MC205263, Origene) or the pcDNA 3.1+ plasmid containing the cloned zfAno1. Materials were diluted at the following ratios, 1.5 µg DNA: 250 µl optimem (Gibco, 31985) and 3µl lipofectamine 2000: 250µl optimem per well. Three wells were transfected per group. Experiments were performed after the cells were cultured in the dish for a minimum of 20h. The experimenter was not blinded to groups.

For efflux assay, 1 ml of cells with a density of ~125,000 cells/ml were dispensed into a 24-well plate and cultured at 37 °C for four hours. Cells were transfected with lipofectamine 2000 per manufacturer’s instructions. For the flux assay, the cells were transfected with an empty pcDNA3.1+ plasmid containing no insert, a pCMV6 plasmid containing mouse Ano1 (NM_178642, Cat # MC205263, Origene) or the pcDNA 3.1+ plasmid containing the cloned zebrafish ano1. Six wells were transfected per group). Materials were diluted at the following ratios, 1 µg DNA: 50 µl optimem and 5µl lipofectamine 2000: 50µl optimem per well. Experiments were performed after the cells were in culture for a minimum of 20h. The experimenter was not blinded to groups.

*Imaging of Ano1*

For examination of Ano1 immunoreactivity, CHO-K1cells were washed with DPBS with calcium and magnesium (Gibco, 14040) and fixed with acetic acid: ethanol (1:3) for 30 seconds. Cells were blocked in PBS (Kodak) with 10% NGS (Fisher, 10000C) and 0.1% triton-X (Fisher, BP151), stained using a rabbit anti-human Anoctamin 1 antibody (Abcam, ab53212) diluted at 1:100 in PBS with 1% NGS. Antibody staining was visualized using an Alexa 488 conjugated goat anti-rabbit secondary antibody (Fisher, A-11008), diluted at 1:1000. Cells were cover slipped with Vectashield containing DAPI (Vector Laboratories, H200010). For examination of GFP signal, coverslips washed with PBS and inverted on slides.

Chemicals were purchased from Fisher Scientific (Pittsburgh, PA) except when otherwise stated. They were sulfuric acid 96% (CAS 7664-93-9), arsenic (III) trioxide (oakwood chemicals, 1327-53-3), ammonia Ce (IV)-sulfate (oakwood chemicals, CAS 10378-47-9), ammonium hydroxide 25% (CAS 1336-21-6), ammonium chloride (CAS 12125-02-9), sodium iodide (CAS 7681-82-5), calcium chloride dihydrate (CAS 10035-04-8), sodium phosphate monobasic (CAS 10049-21-5), magnesium chloride (CAS 7786-30-3), potassium iodide (7681-11-0), sodium hydroxide (CAS 1310-73-2).

*Iodine Flux Assay*

An Iodine Flux Assay was conducted as described by Tang and Wildey (Tang & Wildey, 2004).

*Solutions*

Iodine-loading buffer consisted of the following components: 150 mM NaI, 2 mM CaCl2, 0.8 mM NaH2PO4, 1 mM of MgCl2, and 5.4 mM of KI; adjust pH to 7.4 with 1 N sodium hydroxide.

Detection buffer I was prepared by dissolving 19.8 g of arsenic (III) acid in a mixture of 300 ml-deionized water and 50 ml of 25% ammonia. Sulfuric acid was added to adjust pH to 8.5. Another 28 ml of sulfuric acid and 25 g of ammonium chloride were added to the solution. The final volume was adjusted to 1000 ml with deionized water. Then to slow reaction time, the solution was diluted from a concentration of 100 mM, as used by Tang and Wildey, to 15 mM with deionized water.

Detection buffer II was prepared by dissolving 10 g of ammonia Ce (IV)-sulfate in 474 ml of deionized water. Twenty-six milliliters of sulfuric acid were then added, bringing the final volume to 500 ml. Iodine-loading buffer consisted of the following components: 150 mM NaCI, 2 mMCaCl2, 0.8 mM NaH2PO4, 1 mM of MgCl2, and 5.4 mM of KI; adjust pH to 7.4 with 1 N sodium hydroxide.

Cell lysis buffer consisted of 1% triton X-100 in DPBS.

*Assay reaction time and standard curve generation*

Sodium iodide was dissolved in water to a final concentration of 1.3 mM. A serial dilution of 1:3 was performed in 96-well plates to final concentrations of 1300, 433, 144, 48, 16, 5.3, 1.8, 0.59, 0.2, 0.07, 0.02, and 0.007 μM. In a 96-well plate, 100 microliters of sample was added to each well (n=3), followed by the modified Sandell-Kolthoff reaction as described below. OD405 was measured after the mixture was incubated at room temperature for 10, 15, 20, 25 and 30 min. We examined the OD405 versus concentration curves and selected 15-minute incubation time for subsequent experiments.

*Iodine efflux assay*

A modified protocol for measuring iodine efflux rate was used. 20 hours following transfection, medium was removed, and 1 ml of iodine-loading buffer was added, and cells were incubated for 4 hours at 37 °C. Incubation buffer was removed, and cells were placed into 500 μl of DPBS (with calcium and magnesium and 5% DMSO) with or without 100uM ionomycin (Thermoscientific, CAS # 56092-82-1) for 30 min at 37 °C. Cells were washed two times with 1 ml DPBS containing calcium and magnesium. Cells were lysed with 250 μl of cell lysis buffer and Iodine concentrations were measured with the modified Sandell-Kolthoff (SK) reaction. Typically, 250 μl of lysed sample which contained different concentrations of iodine were mixed with an equal volume of Detection Buffer II and to this was added 250 μl of Detection Buffer I. Solutions were applied in time across, not within groups.

The mixture was incubated at room temperature for 15 min before the OD405 reading with Synergy HTX microplate reader (Biotek). The data presented are raw absorbance readings and have not been normalized or adjusted. The experimenter was not blinded to groups.

*Statistical Analysis*

We utilized GraphPad Prism software to conduct statistical analyses for assessing differences among experimental groups. Specifically, for the flux study analyzing the effect of plasmid we applied Bartlett’s and Forsyth’s tests to evaluate the homogeneity of variances across groups, ensuring that assumptions for subsequent analyses were met. Following this, we performed a Kruskal Wallis test for overall significant differences among group means. When significant results were found, we conducted Dunn's post hoc tests to identify specific group differences. For the flux study evaluating the effect of ionomycin, we used the Shapiro-Wilk test to assess normality and, for the normally distributed data, applied an independent samples t-test to compare differences between two groups. These analyses enabled us to accurately assess and interpret variations within and across experimental conditions.

## Reagents

**Table:** 

**STRAIN**	**GENOTYPE**	**AVAILABLE FROM**
Ab/Ab	Danio Rerio	ZIRC
		
**PLASMID**	**GENOTYPE**	**DESCRIPTION**
pcDNA3.1+	empty	Invitrogen, V79020
pcDNA3.1+	zfAno1	Genebank PQ62605 insert in between XbaI and KpnI
pCMV6	mAno1	NM_178642, Cat # MC205263, Origene
		
**ANTIBODY**	**ANIMAL AND CLONALITY**	**DESCRIPTION**
anti-human Anoctamin 1 antibody	Rabbit polyclonal	Abcam, ab53212, diluted 1:100
Anti-Rabbit IgG (H+L) Cross-Adsorbed Secondary Antibody, Alexa Fluor™ 488	Goat Polyclonal	Fisher, A-11008, diluted at 1:1000.
